# Social connection and cognitive health: A review of concepts, measures, and research priorities

**DOI:** 10.1002/alz.71517

**Published:** 2026-05-26

**Authors:** Reed DeAngelis, Lindsey Burnside, Darby O Donnell, Margaret Hicken, Debra Umberson, Brea Perry

**Affiliations:** ^1^ Institute for Social Research University of Michigan Ann Arbor Michigan USA; ^2^ Department of Sociology University of Texas at Austin Austin Texas USA; ^3^ Department of Sociology and Irsay Institute Indiana University Bloomington Indiana USA

**Keywords:** Alzheimer's disease and Alzheimer's disease and related dementias (AD/ADRD), cognitive health, life course, loneliness, social connection, social isolation

## Abstract

Lack of social connection is a major risk factor for Alzheimer's disease and related dementias, on a par with other common risk factors like physical inactivity and smoking. Yet we still know surprisingly little about how social connection affects cognitive health. Our review provides updated guidance for researchers interested in testing new mechanistic models of social connection and cognitive health over the life course. First, we conceptualize social connection and its various components. Second, we provide readers with a supplemental compendium of social connection measures and discuss how to tailor different types of measures to specific research goals. Third, we advance a conceptual model linking social connection to cognitive health across different social, spatial, and temporal contexts, while also considering potential sources of confounding and reciprocal causation. Finally, we outline five priority areas for future research into social connection and cognitive health across the life course.

## Highlights

- Mechanisms linking social connection to cognitive health are understudied.
- Our review provides new conceptual and measurement guidance for identifying such mechanisms.
- We also provide a supplemental compendium of 79 validated social connection measures.

## 1 INTRODUCTION

Nearly 7 million adults who are 65 years of age and older currently live with Alzheimer's dementia in the United States, and this number is projected to double by the year 2060.^1^ Moreover, those with lower socioeconomic status (SES), who live in rural and small‐town areas, or who identify with a historically marginalized racial‐ethnic group experience higher dementia prevalence relative to their high‐SES, metropolitan, and non‐Hispanic White counterparts, respectively.^1^, ^2^, ^3^ Cognitive health disparities like these suggest that modifiable social‐environmental risk factors play an important role in dementia onset and progression.^4^

Researchers, clinicians, and policymakers increasingly cite *lack of social connection* as one critical social‐environmental risk factor for dementia.^4^, ^5^ Indeed, a new report from the Lancet Commission determined that social isolation in old age likely accounts for more dementia cases than common behavioral risk factors like physical inactivity, smoking, obesity, and hypertension.^4^ Still, another recent review concluded that research on the links between social connection and cognitive health has produced largely conflicting results, owing to inconsistent conceptualizations, measurements, and approaches to sampling and modeling.^5^

Given the current state of the literature, the goal of our review is to address four enduring questions for researchers interested in studying the effects of social connection on cognitive health. First, how should researchers *conceptualize* social connection or the lack thereof? Second, how should researchers *measure* social connection? Third, what are the most relevant *mechanisms, moderators, and confounders* that might condition the effects of social connection on cognitive health? This question also covers issues with reverse causation, or cases when diminished cognitive function might lead to increased social isolation. Fourth, what are the key priority areas for *future research* into social connection and cognitive health?

The following section starts by defining social connection, broadly, before identifying and conceptualizing several of its key components. Our discussion then pivots to the strengths and limitations of different measurement and modeling strategies, which focuses on a measurement compendium constructed by the authors and provided to readers in the online supplement. Drawing from prior empirical research, we then advance a conceptual model linking social connection to cognitive health across different social, spatial, and temporal contexts, while also considering issues of confounding and reverse/reciprocal causation. We end our review by outlining five avenues for future research on social connection and cognitive health.

## 2 WHAT IS SOCIAL CONNECTION?

We use the term “social connection” to reference a multifactorial construct that “encompasses the structure, function, and quality of social relationships.”^6^ ^(p193)^ The *structural* component of social connection refers to the presence, number, and/or interconnectedness of social ties and roles. Structural features of social connection are typically quantitative in nature and include attributes such as the number and interrelatedness of our social ties, number of group memberships, frequencies of interaction with other people and groups, as well as living arrangements (e.g., married/cohabiting or living alone). In the specific context of social networks, structural features refer more narrowly to the configuration of relationships among actors in a bounded network and include measures like density, centrality, structural holes, and network size.^7^

The *functional* dimension includes the resources we receive from social connections. These resources can be potential or actual. The term *social capital* is often used to reference the latent stock of resources embedded within individual social networks or broader social groups, organizations, institutions and communities.^8^, ^9^ At the individual level, for example, someone who is connected to more people in professional occupations or with higher levels of education is said to have “high social network capital,” and is thought to be well‐situated to derive valuable, health‐relevant information from the collective expertise of their social ties.^10^ The resources embedded within social networks become actualized whenever members: (1) exchange emotional (e.g., shows of affection), material (e.g., money), or informational (e.g., problem‐solving guidance) support; (2) influence each other's health behaviors, such as through modeling healthy practices; or (3) derive psychosocial benefits from relationships, such as feeling loved or supported.^6^

Finally, the *quality* dimension of social connection refers to the positive and negative affective features of our relationships.^6^ Examples of positive affective features of social connections include feelings of love, intimacy, and satisfaction with our relationships. Examples of negative affective features include feelings of conflict, strain, and/or overload tied to social relationships and role obligations.

## 3 HOW IS SOCIAL CONNECTION MEASURED?

We now turn to measurement issues. Our discussion centers around the three conceptual components of social connection mentioned earlier: structural, functional, and quality.^6^ We also offer general guidance for readers interested in developing measurement models of social connection. Although space limitations preclude a comprehensive discussion, we highlight key measures here and refer interested readers to a measurement compendium in the online supplement. The compendium includes names, citations, and download links for 77 social connection measures, as well as their units of analysis, target populations, theorized constructs, and the wordings and response options for all indicators comprising multi‐item scales.

### 3.1 Structural measures

Structural measures of social connection reflect the presence and/or number of distinct social ties, as well as the frequency of social interaction and participation in various activities.^6^ We focus here on one of the most common structural measures of social connection: the Social Integration Index (SII).^11^, ^12^ Although different variants have been developed over decades, the SII typically asks respondents to report some or all of the following characteristics: (1) marital/cohabiting status; (2) friend count; (3) frequency of contact with friends, parents, children, and/or neighbors; (4) frequency of volunteering; (5) frequency of religious attendance; and (6) frequency of other group meetings.^11^, ^13^

Like any measure, the SII has unique strengths and limitations. In terms of strengths, the SII is relatively short, easy to administer and score, and covers a wider array of social connections and interactions than many tools, with relatively little burden to participants. The SII can also be adapted to accommodate different age groups.^11^ Even a single‐item indicator from the SII—whether respondents live alone—has been shown to account for significant variation in cognitive health and mortality.^14^

Still, the SII does not account for subjective aspects of social connection, or the potential benefits or resources derived from social ties. The SII also excludes peripheral relationships, like workplace and neighborhood ties,^15^ as well as daily mundane social interactions in public spaces.^16^ In addition, this measure does not account for the structure of relationships among different interconnected people, which often determines the availability and flow of resources and information.^7^ Common to all survey measures discussed here, the SII is also subject to memory recall biases, which could be problematic for studies of cognitive health.^9^ ^(p304)^ We revisit concerns over self‐report biases when we discuss future research priorities.

### 3.2 Functional measures

Functional measures of social connection typically assess the resources people exchange among their social ties. Resources can be either tangible information and support, like physician recommendations and financial aid, or other intangibles like emotional support and guidance.^6^ Thus, functional measures of social connection vary in their degrees of objectivity and subjectivity. On the one hand, asking respondents to report the education, incomes, or occupations of social ties serves as a relatively objective proxy of the socioeconomic resources embedded within their networks. On the other, asking whether or how often respondents feel they can call on their social ties to provide different types of support is inherently more subjective.

One of the most common ways to elicit social resources embedded within a respondent's social networks is the Position Generator (PG) scale, which was designed specifically to measure social network capital.^17^, ^18^ The PG scale presents respondents with a list of occupations and asks them to identify whether any of their family, friends, or acquaintances have jobs like those listed. Similarly, the Resource Generator (RG) scale asks more targeted questions about whether any of the respondent's family, friends, or acquaintances can provide unique resources, skills, or knowledge as needed. Examples include lending large sums of money, helping with moving, discussing intimate matters, playing an instrument, speaking or writing in a foreign language, and/or providing job references (see supplement).

Other measures are better suited for evaluating anticipated socioemotional support from social ties. For instance, the national Midlife in the US (MIDUS) study asks respondents how much they feel their family members and/or spouse care about them, understand how they feel about things, can be relied on for help with a serious problem, and can be opened up to about different worries. Likewise, the Perceived Social Support Module from the National Social Life, Health, and Aging Project (NSHAP) asks respondents how often they can “open up to” and “rely on” family, friends, and their spouse/partner (see supplement).

Scales have also been developed to assess giving support to others. For example, the 2‐Way Social Support Scale asks how often respondents are “there to listen to other's problems” or “help others when they are too busy to get everything done.”^19^ The NSHAP Social Support‐Giving Scale asks respondents similar questions about how often, for instance, “members of your family open up to you if they need to talk about their worries” or “your friends rely on you for help if they have a problem” (see supplement).^20^

Functional scales like these supplement structural measures by providing information not just about the presence or quantity of ties, but also the types of resources people exchange among their social ties. Still, these measures also have some key limitations. For example, embedded in the PG scale are questionable assumptions about the perceived prestige and resources associated with different kinds of occupations, as well as the degree of accessibility of socioeconomic resources through social ties.^17^ The mechanisms linking indicators from the PG and RG scales to respondent health outcomes are also opaque and require careful theorizing, an issue we return to in sections to follow.^9^ ^(p299)^ Finally, neither of these measures capture the more subjective feelings of love, intimacy, and satisfaction with our relationships, or the lack thereof.

### 3.3 Quality measures

Quality measures provide information about the affective aspects of social connection, including perceived levels of conflict, harmony, and (dis)satisfaction with relationships. That is, although structural and functional measures capture the *presence* and *quantity* of different relationships, interactions, and support resources, quality measures attempt to target internalized *feelings* or *attitudes* about these social ties and resources.

One commonly used quality measure is the UCLA Loneliness Scale. Respondents report perceived deficiencies in the quality of social relationships, including feeling as if “nobody really knows them well,” they “lack companionship,” others are “around them but not with them,” or they are “no longer close to anyone,” among other items. In addition, the Interpersonal Mattering Scale gauges how much a respondent feels that others notice and value their presence and view them as an influential person. Example items include “there are people in my life who react to what happens to me in the same way they would if it had happened to them,” and “most people do not seem to notice when I come or when I go.”^21^ Finally, several longitudinal cohort studies like NSHAP, MIDUS, and the Health and Retirement Study (HRS) include harmonized measures of social strain and conflict. These items ask how often family, friends, and spouse “criticize,” “make too many demands,” “let down,” and “get on [the] nerves” of respondents (see supplement).

As these items indicate, quality measures of social connection provide critical information about whether or to what extent respondents feel satisfied with the current state of their relationships, over and above the mere presence or quantity of social ties, interactions, and available resources. However, because these scales rely more heavily on subjective and emotional appraisals, some researchers may be concerned with possible confounding by other individual traits related to personality and mental health.^22^, ^23^, ^24^ In isolation, quality measures also provide little information about different resources or opportunities for interactions that provide more direct cognitive stimulation, independent of how people feel about their social relations.

### 3.4 Multicomponent measures

Given the unique strengths and limitations of structural, functional, and quality measures, some studies and survey instruments attempt to combine features of all three components into holistic assessments of social connection, in efforts to provide a fuller assessment of mechanisms by which social connection may influence cognitive health. One example of such an instrument is the PhenX Social Network Battery (SNB).^25^ The SNB employs an egocentric (i.e., personal) social network approach that centers a focal individual, the significant people in their lives, and the ties among them. What makes this methodological approach distinct is its explicit attention to the structure of interpersonal relations, namely the systematic collection of data on both specific network members and their ties to one another.^7^

Network‐based data provide several key advantages over more global or proxy indicators of social connection. These include improved measurement validity and reliability; the capacity to characterize and incorporate both extensive structural features (e.g., network size, density), as well as compositional measures of function and quality (e.g., percent kin, percent with a college degree, social support, strength of ties); and enhanced analytic flexibility to generate targeted measures for testing competing hypotheses about social mechanisms.^7^, ^26^, ^27^, ^28^, ^29^ However, a disadvantage of the SNB is its high respondent burden and length of administration (roughly 20 min), which is prohibitive for many national surveys.

Longitudinal cohort studies like NSHAP, MIDUS, HRS, and the National Longitudinal Study of Adolescent to Adult Health (Add Health) also include many scales that researchers could combine to evaluate different components of social connection.^11^ Other common scales either include multiple indicators that may capture distinct components when disaggregated from the scale, or single indicators that could be interpreted as reflecting multiple components. For an example of the latter case, the Berkman‐Syme Social Network Index (SNI) and Integrated Questionnaire for the Measurement of Social Capital asks participants to report how many close friends they have, where “close friends” are defined as people respondents “feel at ease with, can talk to about private matters, or call on for help” (see supplement). This single item could be interpreted as gauging structural (“how many close friends”), functional (“call on for help”), and quality (“feel at ease with”) components of connectedness. Although this item efficiently captures multiple facets of social connection, it limits the ability to isolate individual mechanisms. This exercise is meant to highlight why researchers should carefully consider the theoretical mechanisms linking social connection to their outcomes of interest before selecting measures. The following section explores this theme in fuller detail.

### 3.5 General measurement modeling strategies

We now turn to more fundamental measurement issues for readers interested in applying multiple measures and components of social connection in a single study.^30^, ^31^ One aspect of social connection we allude to throughout this article is its high level of abstractness. In measurement modeling terms, we can say “social connection” is a multidimensional *latent* construct that is not directly observable, but rather inferred from other observable indicators like living alone, spending time with social ties, and receiving support or resources from social ties, among numerous others.^30^, ^32^ From this perspective, clarifying how observable indicators are potentially linked with the latent construct of social connection is important.

Three of the most common types of indicators are composite, reflective, and causal.^31^ Each indicator assumes different causal relations with the latent construct. Items comprising popular scales, such as the Social Integration Index (or SII), are examples of composite indicators. Scales like these assume the indicators constitute the latent construct (“social integration”) according to an exact linear function, with equal weights and without measurement error. Although common, these assumptions are often violated in practice.^31^, ^32^

For these reasons, researchers may opt to explicitly model the relationships between latent constructs, observable indicators, and their respective error terms using confirmatory factor analysis (CFA) or structural equation modeling (SEM) frameworks. Describing the mechanics of CFA or SEM is beyond the scope of this work. Instead, we focus on the fundamental logics of reflective and causal indicators. In basic terms, researchers who assume their items are *manifestations of* or *causally dependent upon* a latent construct are employing the logic of reflective indicators. Alternatively, researchers who assume their items are *determinants of* or *precursors to* the latent construct are using the logic of causal indicators.^31^

One initial question to consider is: Does social connection *cause* changes in the indicators, or vice versa? The answer to this question can inform whether you are working with causal or reflective indicators. Although statistical tests have been developed to help adjudicate this question, choosing between causal and reflective indicators requires theory and substantive knowledge.^31^ To explicate these distinctions, we can consider some examples using common measures of social connection. For instance, revisiting the SII, does it make sense to assume that social connection causes changes in a person's marital status, friend count, or frequency of contact with social ties? In this case, the opposite appears true: people are socially connected *because* they are married, have friends, and visit their social ties regularly. That is, we can reasonably assume these measures are causal indicators of social connection.

**FIGURE 1:**
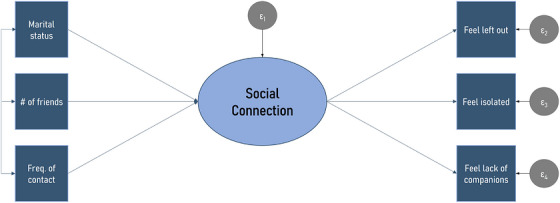
Example of a multiple‐indicator multiple‐cause (MIMIC) model of social connection.

Then what do reflective indicators of social connection look like? One plausible set of reflective indicators includes measures of loneliness. For example, certain questions on the UCLA Loneliness Scale ask respondents how often they feel left out, isolated from others, or that they lack companionship. When combining these items with the Social Isolation Index, we can imagine a process whereby lacking friends or an intimate partner *causes* a person to become socially isolated (i.e., to lack connection), which is ultimately *reflected* in feelings of loneliness.^33^ This process is depicted in Figure 1 as a multiple‐indicator multiple‐cause (MIMIC) latent variable model.^31^ Figure 1 is just one of many possible measurement models that a researcher could develop. The main purpose of this figure is to illustrate how to conceptualize links between observable indicators and the latent construct of “social connection” using previously validated survey scales.

Deciding whether to test measurement models like these will ultimately depend on one's research questions and hypotheses. Researchers should carefully consider the hypothesized mechanisms linking social connection to cognitive health, and whether building a CFA or SEM is preferable for testing these mechanisms. For example, if reduced stress from socioemotional support is the assumed mechanism linking social connection to cognitive health, then building a measurement model with indicators for frequency of contact with close friends and family may be most relevant.^34^, ^35^ If the assumed mechanism is access to health‐relevant information, then the socioeconomic characteristics of social ties may be more relevant.^10^

The same indicators may also reveal unique associations with cognition measures for different groups. For instance, one recent study found that the associations between measures of subjective well‐being and a version of the SII varied by race‐ethnicity and gender.^13^ These findings suggest that mechanisms linking social connection to cognitive health may differ across socioeconomic contexts, a theme we revisit below. Depending on the hypothesized mechanisms, some indicators of social connection may also be more vulnerable than others to issues of reverse causality. For example, the literature reviewed below indicates that older‐age adults tend to disengage from organizations as their cognitive health declines over time, suggesting reverse causality between cognition and social connection.^36^ However, this may be less of an issue for a study testing family support as the key social mechanism of cognitive health among older‐age adults.

### 3.6 Measurement takeaways

To recap, researchers wanting to measure social connection and its links with cognitive health should consider a set of starting questions. First, *what are the hypothesized mechanisms linking social connection to cognitive health*? To organize thinking around mechanisms, we review empirical literature and develop a conceptual model below that links aspects of social connection to cognitive health via several distinct pathways. Second, *which component(s) of social connection is most relevant to your hypothesized mechanism(s)*? You could choose from dozens of structural, functional, and quality measures catalogued in our online supplement.

Third, *who is your target population*? Our measurement compendium includes scales developed for populations as varied as rural older‐age adults (e.g., Wenger Support Network Typology), adult stroke survivors (e.g., Stroke Social Network Scale), homebound adults with clinical depression (e.g., Social Engagement and Activities Questionnaire), university students (e.g., Rochester Interaction Record), working adults (e.g., Workplace Isolation Scale), adults in intimate relationships (e.g., Relationship Closeness Inventory), and even grade‐school children (e.g., Children's Loneliness and Social Dissatisfaction Scale). Fourth, *what is your unit of analysis*? Nearly all scales discussed here are recorded at individual levels. However, a few scales can be measured initially through individual survey responses, and then aggregated at higher social‐spatial levels like social networks and neighborhoods (e.g., Collective Efficacy Scale).

Fifth, assuming you are fielding an original survey, *how much time can you devote to measuring social connection*? You could choose large multi‐component instruments (e.g., PhenX SNB), or single items like living alone and other similar indicators included in the Campaign to End Loneliness Scale, for instance. You may also want to consider shortened versions of common scales. For example, although the original UCLA Loneliness Scale contained 20 items, researchers have validated shorter versions with as few as 3 items.^37^

## 4 CONCEPTUAL FRAMEWORK OF SOCIAL CONNECTION AND COGNITIVE HEALTH

**FIGURE 2:**
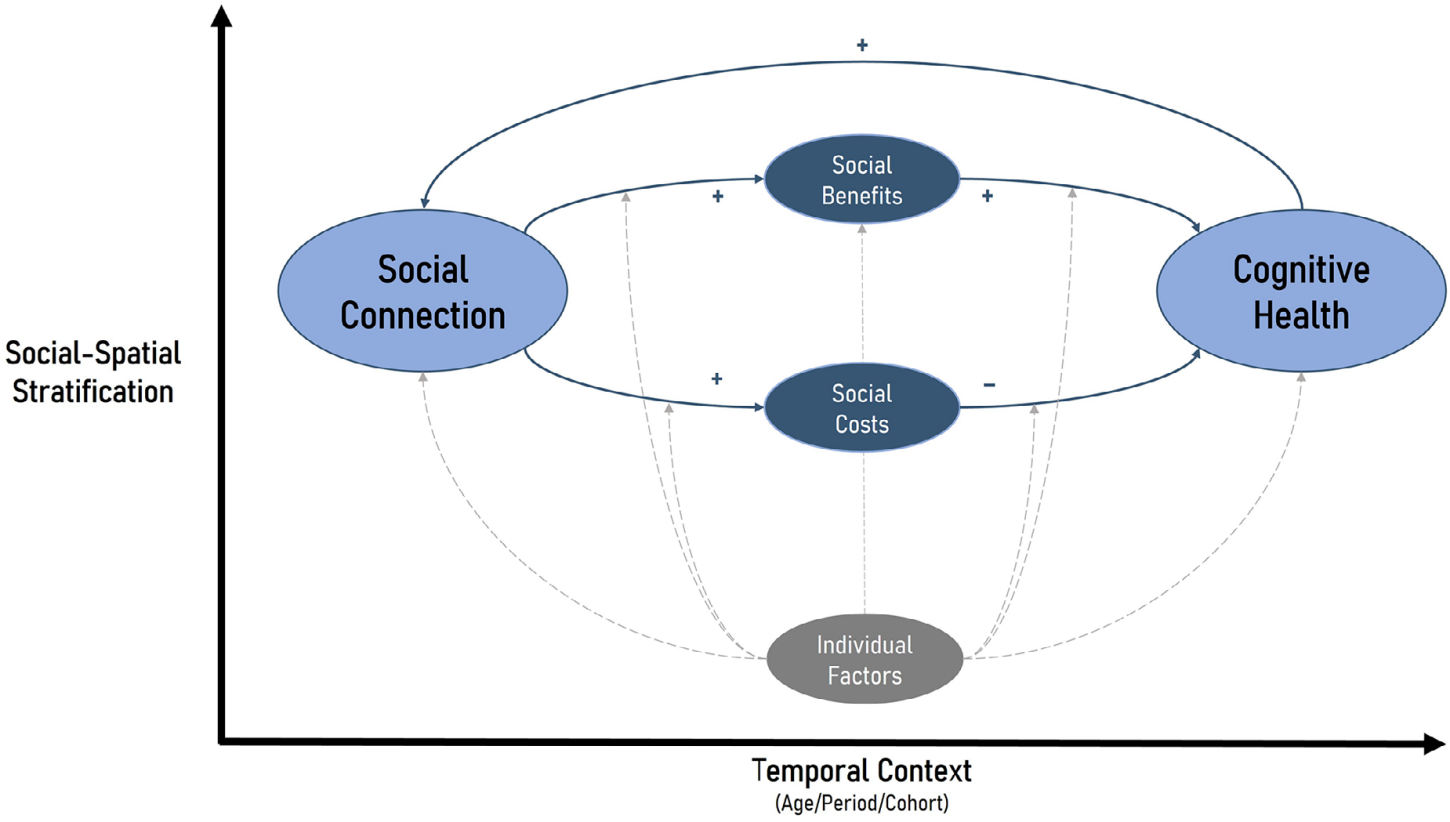
Conceptual model of social connection and cognitive health.

So far, we have discussed different conceptualizations and measurements of social connection. Now we turn to the empirical literature to summarize links between various measures of social connection and cognitive health at the individual level. Our summary of the literature is synthesized into a conceptual framework depicted in Figure 2. As we illustrate in more detail to follow, our framework is grounded in the basic tenets of life course demography and epidemiology, which conceptualize population variation in health and social exposures across distinct times, places, life course stages, and socioeconomic contexts.^38^, ^39^

In this section, we start by highlighting four broad takeaways from Figure 2. The sections that follow provide additional context and evidence for the different pathways depicted in Figure 2. First, the net effects of social connection on cognitive health often reflect a balance between various benefits and costs of social relationships.^40^ Second, we assume everyone is situated within a unique historical period, life course stage, place, and socioeconomic position. These broader social, spatial, and temporal contexts, in turn, jointly influence individual capacities to form and derive cognitive benefits from social connections. Third, we acknowledge that individual‐level factors, such as stable personality traits, may also condition the links between social connection and cognitive health over the life course. Fourth, cognitive health can also affect a person's ability to form and maintain social connections in the first place.

### 4.1 Social connection can enhance cognitive health via social benefits

Social connection can enhance cognitive health by conferring social benefits. Here, we define “social benefits” broadly as any health advantages individuals enjoy by virtue of their social connections.^40^ We highlight five overarching social benefits, which are meant to be illustrative rather than exhaustive.

First, social connection can provide *direct cognitive enrichment*.^41^, ^42^ Interacting regularly with social ties may enhance cognitive health by exposing individuals to novel ideas, activities, and interpersonal cues from body language, facial expressions, and speech patterns.^34^ But whether people derive cognitive enrichment from social connection appears to depend upon the structure and composition of their social networks. For instance, a recent study of older adults in Indiana found that respondents who reported larger social networks comprising mostly loosely connected, non‐kin ties reported better global cognitive function, episodic memory, and executive function, relative to peers with smaller and closer‐knit networks.^34^ These findings underscore the importance of exposure to complex social stimuli from diverse groups of people.

Second and relatedly, social connection also offers novel opportunities for *social and civic engagement*. ^41^, ^43^, ^44^ For instance, a study of older adults in Texas found that participants who reported frequent encounters with diverse social ties typically spent less time sedentary and engaged in a wider variety of activities like socializing and volunteering. Participants involved in more of these activities also tended to report more positive emotions like contentment, calmness, and feeling loved.^44^ In turn, other studies have linked similar activities, especially regular volunteering, to better cognitive health among older‐age adults.^45^, ^46^

Third, social connections can provide *instrumental and emotional supports* that buffer the effects of stressors on brain health.^47^, ^48^, ^49^, ^50^, ^51^ As already discussed, examples of instrumental support include tangible and practical aid like money, transportation, and personal or medical care.^51^ Examples of emotional support include interpersonal shows of sympathy, encouragement, and love.^35^ Numerous studies have found that receiving or being able to anticipate instrumental and emotional support from our social ties, in turn, can boost individual coping capacities and, ultimately, reduce the effects of chronic stress on cognitive health.^41^, ^52^, ^53^, ^54^, ^55^ For instance, a recent systematic review found that people who perceive to have fewer supportive ties tend to report more depressive symptoms over time, which could have implications for cognitive aging.^56^ Although less studied, some theories and emerging evidence also suggest that being able to provide support and maintain a strong sense of mattering to others may generate similar cognitive health benefits by buffering feelings of loneliness and related neurophysiological stress.^57^, ^58^, ^59^

Fourth, our social connections also enforce norms and obligations that can encourage *regulation of healthy behaviors*. For example, married persons and parents with children in the home have been found to report healthier lifestyles and fewer cardiovascular risk factors (e.g., less substance use), relative to their single and childless peers.^60^, ^61^ In these contexts, the roles of parent and spouse entail unique responsibilities and commitments that can motivate a person to take better care of themselves.^62^ Other social ties like friends and neighbors can also reinforce positive health norms like physical activity, avoidance of nicotine, moderate alcohol use, healthy diet, and regular doctor visits, among others.^63^, ^64^ Maintaining healthy behaviors, especially regular physical activity and healthy diet, has been shown to predict better cognitive health and slower rates of cognitive decline in old age.^65^, ^66^

Fifth, social connections can also provide *information about health care providers* that we may be unable to access on our own. One national study of U.S. adults found that those who reported more social ties with higher average levels of education tended to seek clinician referrals and other health information more often and from more diverse sources, relative to peers with fewer and lesser educated ties. These patterns persisted regardless of the respondent's age, gender, race‐ethnicity, marital status, education, occupation, or income.^10^ Parallel studies have found that health care access, in turn, may promote cognitive health.^67^

### 4.2 Social connection can harm cognitive health via social costs

Increased social connection can also harm cognitive health via social costs. Broadly, “social costs” refer to any health damages individuals experience by virtue of their social connections.^40^ We highlight three general types of social costs, which are meant to serve again as illustrative but non‐exhaustive examples.

First, our social connections can generate chronic stress via *strained social interactions*.^50^, ^68^, ^69^ For example, national cohort studies by Yang and colleagues have found that increased exposure to social strain (e.g., “How much do family and friends criticize you?”) predicts biomarkers of chronic inflammation and cardiometabolic dysregulation across the life course.^11^, ^50^ Pertinent to cognitive aging, a community study of older‐age adults in Chicago found that participants reported faster cognitive decline over a 6‐year period if they also reported more negative interactions with social network ties (e.g., “In the past month, how often did the people you know act angry or upset with you?”). These patterns persisted regardless of initial cognitive function, age, sex, education, frequency of social interaction, depressive symptoms, or loneliness.^70^

Second, social connections can also become *overburdensome and repressive*. For instance, studies have found that while most people believe their social support networks are equitable, those who feel they provide more support than they receive tend to report worse health and less satisfaction with their social networks.^71^, ^72^ Studies have also found evidence suggesting that people who care for children, parents, or a sick spouse exhibit signs of poor mental health and cognitive decline.^54^, ^73^ Another study of the HRS cohort found that people who maintain high levels of religious involvement over the life course tend to exhibit worse cognitive function in older age. The authors of the study speculated that certain religious groups may promote rigid belief systems and lifestyles that constrain opportunities for intellectual discourse and cognitive stimulation.^74^

Third, social connection also increases our risk of *exposure to adverse health influences*.^40^, ^63^, ^64^ Our social networks can expose us to common pathogens and infectious diseases that are known to predict long‐term neurodegeneration.^75^, ^76^ Social networks can also spread health misinformation and influence individuals to engage in self‐destructive behaviors like binge drinking and smoking.^63^, ^64^ For instance, social networks were incubators of misinformation in the early phases of the coronavirus disease 2019 (COVID‐19) pandemic.^77^ People embedded in such networks were at heightened risk of vaccine skepticism and COVID‐19 infection.^78^ COVID‐19 infection, in turn, can lead to neurodegeneration and cognitive decline.^79^

### 4.3 Other individual factors can affect social connection and cognitive health

Up to this point, we have reviewed evidence indicating that different aspects of social connection can either benefit or harm cognitive health. In this section, we briefly consider how other individual‐level factors can influence these same processes. Research on this topic broadly suggests that certain stable personality traits may moderate or confound observed associations between social connection and cognitive health.

For example, a recent meta‐analysis found that people who score higher on personality traits like extraversion or openness to new experiences and ideas tend to exhibit better cognitive health, whereas those scoring higher on neuroticism typically show worse cognitive outcomes.^80^ A separate longitudinal cohort study of older adults in Dublin, Ireland, found that participants who scored higher on extraversion and lower on neuroticism also reported more supportive social ties, on average, than their less extraverted/more neurotic peers. Being more extraverted and reporting more supportive ties, in turn, independently predicted better cognitive function over a 2‐year period.^22^ Another cross‐sectional study of Canadian adults 45–85 years likewise found that increased social isolation and loneliness partially accounted for associations between higher neuroticism scores and diminished cognitive function.^81^ Together, these studies suggest that personality traits may at least partially account for links between social connection and cognitive health.

### 4.4 Cognitive health can affect social connection

Studies reviewed so far have focused on the potential effects of social connection on cognitive health (i.e., social causation). An emerging literature indicates that cognitive health can also affect social connection (i.e., social selection). These findings are important for at least two reasons. First, they suggest the possibility of *reverse causation*, which has implications for inferences researchers can reliably draw from observational data, especially cross‐sectional studies. Second, findings like these also suggest the potential for *reciprocal causation*, or positive feedback loops between social connection and cognitive health over the life course.

For instance, a longitudinal study of older adults in the United States found that participants with declining memory functioning tended to report increasing loneliness over time.^82^ Another longitudinal study of older adults in South Korea found that respondents with declining cognitive function tended to report fewer organizational activities over time.^36^ A third longitudinal study of older adults in Japan found a positive feedback loop between social isolation and functional impairments, whereby those who reported more impairments tended to report increasing social isolation over time and vice versa.^83^

### 4.5 The role of social–spatial contexts

Our conceptual model also accounts for other social, spatial, and temporal contexts that may influence levels of social connection and related effects on cognitive health. In this section, we first unpack social and spatial contexts. The following section will then consider temporal context.

The y‐axis depicts social–spatial contexts as hierarchical stratifying forces that differentially allocate opportunities to form and derive cognitive benefits from social connections. Axes of social stratification include race‐ethnicity, gender, sexual identity, disability status, education, occupation, and wealth, among others.^84^ Although typically proxied at the individual level, we conceptualize axes of social stratification as supra‐individual characteristics of a society that can determine individual‐level exposure to cognitive hazards and access to cognitively enriching resources.^85^, ^86^, ^87^ Interlaced with axes of social stratification are spatial contexts like neighborhoods, cities, states, regions, and nations. To say that axes of social and spatial stratification are “interlaced” is to acknowledge that places where people live, work, and enjoy leisure reflect sites of inclusion, exclusion, and the unequal exercise of social and political‐economic power.^88^, ^89^, ^90^

There are several examples to indicate that broader socioeconomic contexts can mediate or moderate our capacities to form and derive cognitive benefits from social connections. In terms of SES, people with more education and income typically report larger, more diverse, and better resourced social networks than their disadvantaged peers,^8^, ^91^ as well as better cognitive function.^92^, ^93^ However, having a larger and more diverse social network appears to disproportionately benefit the cognitive health of individuals with lower education levels.^94^ In terms of occupational status, a recent study of older‐age adults in Indiana found that participants who worked in jobs involving more complex interactions with other people exhibited better cognitive function, relative to their peers who worked jobs involving fewer complex social interactions.^95^ The transition to retirement has also been found to predict accelerated cognitive decline,^96^ which other studies suggest could be explained by increased risks of social isolation and loneliness.^97^, ^98^

Studies also indicate that certain minoritized racial‐ethnic groups tend to exhibit higher rates of Alzheimer's disease/Alzheimer's disease and related dementias (AD/ADRD) relative to their White counterparts,^3^ as well as more social isolation and other similar social risk factors for AD/ADRD.^99^ For example, studies have found that Black and Hispanic (vs White) adults tend to work in occupations that involve fewer complex interactions with people, which partially accounts for their relatively worse cognitive health outcomes.^100^, ^101^ Another analysis of the NSHAP cohort found that when compared to White peers, Black and Hispanic older‐age adults typically reported smaller social networks, less frequent interactions with social ties, and stronger feelings of loneliness.^102^

Regarding gender and sexual identity, studies find that men usually report more social isolation than women across the life course,^103^ yet the effects of social isolation on cognitive health and longevity appear to be comparable for women and men.^103^, ^104^ Relative to their cisgender and heterosexual peers, people who identify as LGBTQ+ also tend to report fewer reliable sources of social support and increased social isolation across the life course.^105^, ^106^ Moreover, a recent study of the Behavioral Risk Factor Surveillance System found that adults who identified as sexual minorities (i.e., gay, bisexual, or other) were less likely than their heterosexual peers to report being married or receiving needed social support, and more likely to report difficulties with memory, concentration, and performing various activities of daily living.^107^

Spatial contexts at various scales can also determine individual‐level access to social connections,^108^ with potentially critical implications for cognitive health. For instance, different types of social infrastructure can enrich cognition by promoting social interactions at local spatial scales like neighborhoods.^109^, ^110^ Social infrastructure refers to the “networks of spaces, facilities, institutions, and groups that create affordances for social connection.”^111^ ^(p758)^ Examples include parks, libraries, community centers, gyms, coffee shops, sidewalks, high streets, and religious centers.^109^, ^110^ Emerging evidence suggests that inequalities in access to social infrastructure can account for population disparities in cognitive health.^112^, ^113^, ^114^, ^115^

Other features of residential contexts can also facilitate or constrain opportunities to form and derive cognitive benefits from social connections.^116^, ^117^, ^118^, ^119^ For example, neighbors can become part of our social networks, providing instrumental support, health‐relevant information, and opportunities for civic engagement.^117^, ^120^, ^121^ Nevertheless, throughout most of the twentieth century, institutionalized segregation (e.g., redlining) and car‐centric city planning across the United States has generated stark inequalities in local social infrastructures and spatial connectivity that persist into the present era.^122^, ^123^, ^124^ As a result, opportunities to form cognitively enriching connections with neighbors and other community members tend to be concentrated within affluent and predominantly White areas.^112^, ^118^, ^125^ This broader historical context further highlights the inextricable links between axes of social, economic, and spatial inequality with individual cognitive health and broader population health disparities.

### 4.6 The role of time

Time is another major force shaping social connections and their roles in cognitive health. The x‐axis of Figure 2 depicts the passage of time in terms of age, period, and cohort. Age refers to within‐person developmental changes over the life course. Period refers to major events, grounded in calendar years, which can affect every living person regardless of age. Finally, cohort refers to a group of people born in a similar period and thus exposed to similar cultural norms and historical events at the same life course stages.^126^, ^127^, ^128^

Regarding the role of age, evidence generally indicates people tend to report fewer social interactions as they transition through adulthood and into old age, especially after exiting social roles related to childrearing and work.^103^, ^129^, ^130^, ^131^ Yet other evidence indicates that many older adults maintain core support networks and regular contacts with neighbors and religious or volunteer groups.^131^, ^132^ Grandparenting can also offer new opportunities for social interaction and cognitive stimulation in late life. For example, studies of diverse samples find that older adults who occasionally care for grandchildren tend to report better subjective well‐being and cognitive health, relative to their counterparts who provide no or high‐intensity care.^133^, ^134^ However, consistent with our conceptual model, the cognitive benefits of grandparenting appear to depend on broader socioeconomic circumstances, with those in higher status contexts benefitting disproportionately.^135^, ^136^ Patterns like these also underscore how social ties change with age. Although parents and schoolmates often play an outsized role earlier in life, marital partners, co‐workers, (grand)children, and neighbors typically comprise most of our social connections in adulthood.^11^

In terms of period and cohort effects, the COVID‐19 pandemic represents a global period effect that led to prolonged social distancing and, ultimately, increased social isolation for people of all ages and birth cohorts.^137^ Studies also find that health inequalities between married and single persons have converged in recent decades, presumably due to evolving cultural norms associated with marriage among younger birth cohorts.^138^ Robert Putnam and colleagues have also famously argued that rates of social and civic engagement have declined among recent birth cohorts, presumably due to increasing cultural emphases on individualism and an increasing reliance on digital communication technologies, among various other reasons.^139^ Yet more recent empirical work suggests that rates of social and civic engagement may not have not declined among younger birth cohorts, but rather evolved into new forms.^140^, ^141^

## 5 CONCLUSION: FUTURE RESEARCH PRIORITIES

Future research should address several enduring questions. First, what are the broader contextual determinants of social connection? We reviewed several articles that have revealed important initial patterns, but more work on this topic is needed.^129^ For instance, emerging evidence suggests that rural and urban residents maintain distinct social network structures, with rural residents reporting more close‐knit networks based largely on kin relations.^142^ Urban greening initiatives, such as the conversion of vacant lots into public green spaces, have also been found to promote social connection among residents.^143^ These are just a couple examples of frameworks and interventions that could help to address this question going forward.

Second, how does digital social connection affect cognitive health? Increasingly, people of all ages rely on digital technologies to stay connected with family, friends, and acquaintances. ^144^, ^145^ Interactions with artificial intelligence (AI) are also becoming more commonplace, which may have additional implications for cognitive health and aging.^146^, ^147^ Researchers have begun to conceptualize and measure real‐world socioeconomic consequences of digital social ties and interactions.^148^ But potential links between digital social connection and cognitive health are understudied.^149^, ^150^ Answers to this question will likely depend on the life course stage of the study population.^146^ For instance, recent evidence from Japan suggests that older adults who are physically isolated report better health if they also use smartphone devices regularly to maintain digital social connections.^83^ A new meta‐analysis of 57 observational studies of older‐age adults also found that reported use of digital technologies predicted significantly reduced odds of cognitive impairment and slower rates of cognitive decline.^151^ However, regular social media use appears to be more problematic for the emotional and cognitive development of children and adolescents.^152^

Third, are there cognitive benefits associated with solitude or minimal social interaction? As discussed earlier, our social ties can become a source of strain and distress in certain contexts.^11^, ^40^, ^50^ Moreover, social isolation does not appear to be innately harmful, evidenced by generally weak correlations between being alone and feeling lonely.^153^ Solitary activities like meditation or solo nature walks may also reduce stress and enhance cognitive functioning.^153^, ^154^, ^155^ One recent study has also found that people who score higher on measures of social anxiety tend to report better mental health from engaging in digitally‐mediated and/or small‐group (vs large‐group) social interactions.^156^ These findings suggest that minimizing social connection may have unique cognitive health benefits, at least in specific contexts or for certain types of people.

Fourth, how does bereavement affect cognitive health? The death of loved ones is among the most profound costs of social connection,^157^ and emerging evidence suggests that bereavement and resulting grief may be important sources of distress and cognitive decline.^158^, ^159^, ^160^ For instance, older‐age adults who experience the death of a spouse tend to report increased feelings of loneliness and social isolation over time.^161^ Studies have also revealed social inequalities in exposure to bereavement. For instance, Black Americans typically report significantly more deaths of loved ones than White peers, and at earlier stages of the life course.^162^, ^163^, ^164^ Together, these findings suggest that bereavement may be an underexplored mechanism linking social connection to cognitive health disparities.

Fifth, how can we improve measurement of social connection? As previously mentioned, research in this area relies heavily on self‐reported survey data, where participants are tasked with reporting social ties and interactions alongside tests of cognitive function. But this strategy could introduce substantial measurement error when a participant's memory and cognition affect their ability to respond to complex survey questions in the first place. For this reason, researchers are innovating new measurement strategies that can infer a participant's daily social interactions using passive sensors.^165^, ^166^, ^167^ Moreover, given that associations between survey items of social connection and health appear to vary for different socioeconomic groups,^13^ future work may benefit from conducting in‐depth focus groups and cognitive interviews to assess how different populations interpret the same survey instruments of social connection.^168^, ^169^ Finally, new multilevel measurement and modeling strategies are needed to measure social connections at broader social–spatial levels like organizations, neighborhoods, cities, and so on.^170^, ^171^ For instance, emerging evidence suggests that living in places where residents tend to trust other people and institutions may have unique cognitive health benefits, over and above the cognitive benefits we derive from our individual social connections.^172^, ^173^, ^174^

## CONFLICT OF INTEREST STATEMENT

The authors declare no conflicts of interest. Author disclosures are available in the Supporting Information.

## Supporting information



Supplementary Information

Supplementary Information

## References

[1] 2024 Alzheimer's disease facts and figures. Alzheimer's Dement. 2024;20(5):3708‐3821. doi:10.1002/alz.13809 38689398 PMC11095490

[2] Ho JY , Franco Y . The rising burden of Alzheimer's disease mortality in rural America. SSM—Population Health. 2022;17:101052. doi:10.1016/j.ssmph.2022.101052 35242995 PMC8886050

[3] Matthews KA , Xu W , Gaglioti AH , Holt JB , Croft JB , Mack D , McGuire LC Racial and ethnic estimates of Alzheimer's disease and related dementias in the United States (2015‐2060) in adults aged ≥65 years. Alzheimer's Dement. 2019;15(1):17‐24. doi:10.1016/j.jalz.2018.06.3063 30243772 PMC6333531

[4] Livingston G , Huntley J , Liu KY , et al. Dementia prevention, intervention, and care: 2024 report of the Lancet standing Commission. Lancet. 2024;404(10452):572‐628. doi:10.1016/S0140-6736(24)01296-0 39096926

[5] Guarnera J , Yuen E , Macpherson H . The impact of loneliness and social isolation on cognitive aging: a narrative review. J Alzheimer's Dis Rep. 2023;7(1):699‐714. doi:10.3233/ADR-230011 37483321 PMC10357115

[6] Holt‐Lunstad J . Social connection as a public health issue: the evidence and a systemic framework for prioritizing the “Social” in social determinants of health. Annu Rev Public Health. 2022;43(1):193‐213. doi:10.1146/annurev-publhealth-052020-110732 35021021

[7] Perry BL , Pescosolido BA , Borgatti SP . Egocentric Network Analysis: Foundations, Methods, and Models. Cambridge University Press; 2018.

[8] Portes A . Social capital: its origins and applications in modern sociology . Annu Rev Sociol. 1998;24:1‐24.

[9] Kawachi I , Berkman LF , Glymour MM , eds. Social Epidemiology. 2nd ed. Oxford University Press; 2015. doi:10.1093/med/9780195377903.001.0001

[10] Song L , Chang TY . Do resources of network members help in help seeking? social capital and health information search. Soc Netw. 2012;34(4):658‐669. doi:10.1016/j.socnet.2012.08.002

[11] Yang YC , Boen C , Gerken K , Li T , Schorpp K , Harris KM . Social relationships and physiological determinants of longevity across the human life span. Proc Natl Acad Sci. 2016;113(3):578‐583. doi:10.1073/pnas.1511085112 26729882 PMC4725506

[12] Berkman LF , Syme SL . Social networks, host resistance, and mortality: a nine‐year follow‐up study of alameda county residents. Am J Epidemiol. 1979;109(2):186‐204. 425958 10.1093/oxfordjournals.aje.a112674

[13] Nguyen ND , Lin Z . Social isolation and subjective well‐being among older adults: a longitudinal examination by race/ethnicity and gender. J Gerontol B Psychol Sci Soc Sci. 2025;80(6):gbaf066. doi:10.1093/geronb/gbaf066 40197526 PMC12105471

[14] Klinenberg E . Social isolation, loneliness, and living alone: identifying the risks for public health. Am J Public Health. 2016;106(5):786‐787. doi:10.2105/AJPH.2016.303166 27049414 PMC4985072

[15] Marshall GW , Michaels CE , Mulki JP . Workplace isolation: exploring the construct and its measurement. Psychol & Marketing. 2007;24(3):195‐223. doi:10.1002/mar.20158

[16] Finlay J , Esposito M , Kim MH , Gomez‐Lopez I , Clarke P . Closure of ‘third places’? exploring potential consequences for collective health and wellbeing. Health Place. 2019;60:102225. doi:10.1016/j.healthplace.2019.102225 31622919 PMC6934089

[17] Marsden PV , Baum DS . Occupational selection and the reliability of position generator measures of social capital. Soc Netw. 2024;79:34‐47. doi:10.1016/j.socnet.2024.05.001

[18] Meanley S , Biernesser C , O'Malley T , Bear T , Trauth J . Employing position generators to assess social capital and health: a scoping review of the literature and recommendations in future population health surveillance. 2020;13(3):16‐45. PMC8552962 34721948

[19] Shakespeare‐Finch J , Obst PL . The development of the 2‐way social support scale: a measure of giving and receiving emotional and instrumental support. J Pers Assess. 2011;93(5):483‐490. doi:10.1080/00223891.2011.594124 21859288

[20] Hawkley L , Wroblewski K , Cagney KA , Waite LJ . Resilience and social support‐giving scales: conceptual and empirical validation. J Gerontol Ser B. 2021;76(Supp 3):S238‐S250. doi:10.1093/geronb/gbab091 PMC8678431 34918150

[21] Elliott G , Kao S , Grant AM . Mattering: empirical validation of a social‐psychological concept. Self Identity. 2004;3(4):339‐354. doi:10.1080/13576500444000119

[22] McHugh Power JE , Lawlor BA , Kee F . Social support mediates the relationships between extraversion, neuroticism, and cognitive function in older adults. Public Health. 2017;147:144‐152. doi:10.1016/j.puhe.2017.02.015 28404490

[23] Shehab I , Nassauer JI , Webster NJ , Sampson N , Li J . Perceptions of Detroit vacant lot greening designs related to depressive symptoms and household flooding. Urban For Urban Green. 2024;96:128358. doi:10.1016/j.ufug.2024.128358

[24] Campos‐Matos I , Subramanian SV , Kawachi I . The ‘dark side’ of social capital: trust and self‐rated health in European countries. Eur J Public Health. 2016;26(1):90‐95. doi:10.1093/eurpub/ckv089 26017573

[25] PhenX Toolkit . Social Networks. Published online 2010. Accessed February 2, 2026. https://www.phenxtoolkit.org/protocols/view/211101

[26] Burt RS . A note on the general social survey's ersatz network density item. Soc Netw. 1987;9(1):75‐85. doi:10.1016/0378-8733(87)90019-0

[27] Cornwell B , Goldman A , Laumann EO . Homeostasis revisited: patterns of stability and rebalancing in older adults’ social lives. J Gerontol Ser B. 2021;76(4):778‐789. doi:10.1093/geronb/gbaa026 PMC7955992 32080742

[28] Bernard HR , Johnsen EC , Killworth PD , McCarty C , Shelley GA , Robinson S . Comparing four different methods for measuring personal social networks. Soc Netw. 1990;12(3):179‐215. doi:10.1016/0378-8733(90)90005-T

[29] Moody J , Paxton P . Building bridges: linking social capital and social networks to improve theory and research. Am Behav Sci. 2009;52(11):1491‐1506. doi:10.1177/0002764209331523

[30] Bollen KA , Hoyle RH . Perceived cohesion: a conceptual and empirical examination. Soc Forces. 1990;69(2):479‐504.

[31] Bollen KA , Bauldry S . Three Cs in measurement models: causal indicators, composite indicators, and covariates. Psychol Methods. 2011;16(3):265‐284. doi:10.1037/a0024448 21767021 PMC3889475

[32] Bollen KA . Structural Equations with Latent Variables. John Wiley & Sons; 1989.

[33] Perlman D , Peplau LA . Loneliness. In: Friedman H.S. , eds. Encyclopedia of Mental Health. Academic Press; 1998:571‐581.

[34] Perry BL , McConnell WR , Peng S , Roth AR et al. Social networks and cognitive function: an evaluation of social bridging and bonding mechanisms. Gerontologist. 2022;62(6):865‐875. doi:10.1093/geront/gnab112 34338287 PMC9290895

[35] Thoits PA . Mechanisms linking social ties and support to physical and mental health. J Health Soc Behav. 2011;52(2):145‐161. doi:10.1177/0022146510395592 21673143

[36] Son J , Sung P . The reciprocal relationship between social engagement and cognitive function among older adults in South Korea. J Appl Gerontol. 2023;42(5):928‐941. doi:10.1177/07334648221148953 36583249

[37] Hughes ME , Waite LJ , Hawkley LC , Cacioppo JT . A short scale for measuring loneliness in large surveys: results from two population‐based studies. Res Aging. 2004;26(6):655‐672. doi:10.1177/0164027504268574 18504506 PMC2394670

[38] Kuh D , Ben‐Shlomo Y , Lynch J , Hallqvist J , Power C . Life course epidemiology. J Epidemiol Community Health. 2003;57(10):778‐783. doi:10.1136/jech.57.10.778 14573579 PMC1732305

[39] Harris KM , McDade TW . The biosocial approach to human development, behavior, and health across the life course. RSF. 2018;4(4):2‐26. doi:10.7758/RSF.2018.4.4.01 30923747 PMC6434524

[40] Song L , Pettis PJ , Chen Y , Goodson‐Miller M . Social cost and health: the downside of social relationships and social networks. J Health Soc Behav. 2021;62(3):371‐387. doi:10.1177/00221465211029353 34309419

[41] Perry BL , McConnell WR , Coleman ME , Roth AR , Peng S , Apostolova LG . Why the cognitive “fountain of youth” may be upstream: pathways to dementia risk and resilience through social connectedness. Alzheimer's Dement. 2022;18(5):934‐941. doi:10.1002/alz.12443 34482619 PMC8897512

[42] Perry BL , McConnell WR , Peng S , et al. Social networks and cognitive function: an evaluation of social bridging and bonding mechanisms. Gerontologist. 2022;62(6):865‐875. doi:10.1093/geront/gnab112 34338287 PMC9290895

[43] Lewis VA , MacGregor CA , Putnam RD . Religion, networks, and neighborliness: the impact of religious social networks on civic engagement. Soc Sci Res. 2013;42(2):331‐346. doi:10.1016/j.ssresearch.2012.09.011 23347480

[44] Fingerman KL , Huo M , Charles ST , Umberson DJ . Variety Is the spice of late life: social integration and daily activity. J Gerontol Ser B. 2020;75(2):377‐388. doi:10.1093/geronb/gbz007 PMC7179804 30783671

[45] Sharifi S , Babaei Khorzoughi K , Rahmati M . The relationship between volunteering and cognitive performance in older adults: a systematic review. Geriatr Nurs. 2024;55:89‐96. doi:10.1016/j.gerinurse.2023.10.020 37976560

[46] Kail BL , Carr DC . More than selection effects: volunteering Is associated with benefits in cognitive functioning. J Gerontol Ser B. 2020;75(8):1741‐1746. doi:10.1093/geronb/gbaa101 32696973

[47] House JS , Umberson D , Landis KR . Structures and processes of social support. Annu Rev Sociol. 1988;14:293‐318.

[48] Thoits PA . Stress, coping, and social support processes: where are we? what next? J Health Soc Behav. 1995;35:53. doi:10.2307/2626957 7560850

[49] Berkman LF . Social support, social networks, social cohesion and health. Soc Work Health Care. 2000;31(2):3‐14. doi:10.1300/J010v31n02_02 11081851

[50] Yang YC , Schorpp K , Harris KM . Social support, social strain and inflammation: evidence from a national longitudinal study of U.S. adults. Soc Sci Med. 2014;107:124‐135. doi:10.1016/j.socscimed.2014.02.013 24607674 PMC4028709

[51] Schultz BE , Corbett CF , Hughes RG . Instrumental support: a conceptual analysis. Nurs Forum. 2022;57(4):665‐670. doi:10.1111/nuf.12704 35133664 PMC9544712

[52] Cacioppo JT , Hawkley LC . Social isolation and health, with an emphasis on underlying mechanisms. Perspect Biol Med. 2003;46(3):S39‐S52. doi:10.1353/pbm.2003.0049 14563073

[53] Eisenberger NI , Taylor SE , Gable SL , Hilmert CJ , Lieberman MD . Neural pathways link social support to attenuated neuroendocrine stress responses. Neuroimage. 2007;35(4):1601‐1612. doi:10.1016/j.neuroimage.2007.01.038 17395493 PMC2710966

[54] Umberson D , Karas Montez J . Social relationships and health: a flashpoint for health policy. J Health Soc Behav. 2010;51(supp l):S54‐S66. doi:10.1177/0022146510383501 20943583 PMC3150158

[55] Costa‐Cordella S , Arevalo‐Romero C , Parada FJ , Rossi A . Social support and cognition: a systematic review. Front in Psychol. 2021;12:637060. doi:10.3389/fpsyg.2021.637060 PMC7941073 33708164

[56] Wang J , Mann F , Lloyd‐Evans B , Ma R , Johnson S . Associations between loneliness and perceived social support and outcomes of mental health problems: a systematic review. BMC Psychiatry. 2018;18(1):156. doi:10.1186/s12888-018-1736-5 29843662 PMC5975705

[57] Flett GL , Heisel MJ . Aging and feeling valued versus expendable during the covid‐19 pandemic and beyond: a review and commentary of why mattering is fundamental to the health and well‐being of older adults. Int J Ment Health Addict. 2021;19(6):2443‐2469. doi:10.1007/s11469-020-00339-4 32837430 PMC7295320

[58] Inagaki TK . Neural mechanisms of the link between giving social support and health. Ann NY Acad Sci. 2018;1428(1):33‐50. doi:10.1111/nyas.13703 29749102

[59] Eisenberger NI . An empirical review of the neural underpinnings of receiving and giving social support: implications for health. Psychosom Med. 2013;75(6):545‐556. doi:10.1097/PSY.0b013e31829de2e7 23804014 PMC3763941

[60] Umberson D . Family status and health behaviors: social control as a dimension of social integration. J Health Soc Behav. 1987;28(3):306. doi:10.2307/2136848 3680922

[61] Manfredini R , De Giorgi A , Tiseo R , et al. Marital status, cardiovascular diseases, and cardiovascular risk factors: a review of the evidence. J Women's Health. 2017;26(6):624‐632. doi:10.1089/jwh.2016.6103 28128671

[62] Umberson D . Gender, marital status and the social control of health behavior. Soc Sci Med. 1992;34(8):907‐917. doi:10.1016/0277-9536(92)90259-S 1604380

[63] Smith KP , Christakis NA . Social networks and health. Annu Rev Sociol. 2008;34(1):405‐429. doi:10.1146/annurev.soc.34.040507.134601

[64] Zhang J , Centola D . Social networks and health: new developments in diffusion, online and offline. Annu Rev Sociol. 2019;45(1):91‐109. doi:10.1146/annurev-soc-073117-041421

[65] Erickson KI , Donofry SD , Sewell KR , Brown BM , Stillman CM . Cognitive aging and the promise of physical activity. Annu Rev Clin Psychol. 2022;18(1):417‐442. doi:10.1146/annurev-clinpsy-072720-014213 35044793

[66] Halloway S , Wagner M , Tangney C , et al. Profiles of lifestyle health behaviors and cognitive decline in older adults. Alzheimer's Dement. 2024;20(1):472‐482. doi:10.1002/alz.13459 37676928 PMC10840675

[67] Mullins MA , Bynum JPW , Judd SE , Clarke PJ . Access to primary care and cognitive impairment: results from a national community study of aging Americans. BMC Geriatrics. 2021;21(1):580. doi:10.1186/s12877-021-02545-8 34670519 PMC8527792

[68] Rook KS . Parallels in the study of social support and social strain. J Soc Clin Psychol. 1990;9(1):118‐132.

[69] Krause N , Rook KS . Negative interaction in late life: issues in the stability and generalizability of conflict across relationships. J Gerontol Ser B. 2003;58(2):P88‐P99. doi:10.1093/geronb/58.2.P88 PMC3833830 12646592

[70] Wilson RS , Boyle PA , James BD , Leurgans SE , Buchman AS , Bennett DA . Negative social interactions and risk of mild cognitive impairment in old age. Neuropsychology. 2015;29(4):561‐570. doi:10.1037/neu0000154 25495828 PMC4468039

[71] Chandola T , Marmot M , Siegrist J . Failed reciprocity in close social relationships and health: findings from the Whitehall II study. J Psychosom Res. 2007;63(4):403‐411. doi:10.1016/j.jpsychores.2007.07.012 17905049 PMC2072816

[72] Taylor RJ , Mouzon DM , Nguyen AW , Chatters LM . Reciprocal family, friendship and church support networks of African Americans: findings from the national survey of American life. Race Soc Probl. 2016;8(4):326‐339. doi:10.1007/s12552-016-9186-5 27942269 PMC5142742

[73] Christian LM , Wilson SJ , Madison AA , et al. Understanding the health effects of caregiving stress: new directions in molecular aging. Ageing Res Rev. 2023;92:102096. doi:10.1016/j.arr.2023.102096 37898293 PMC10824392

[74] Hill TD , Carr DC , Burdette AM , Dowd‐Arrow B , Dowd‐arrow B. life‐course religious attendance and cognitive functioning in later life. Res Aging. 2020;42(7‐8):217‐225. doi:10.1177/0164027520917059 32266864

[75] Duggan MR , Peng Z , Sipilä PN , et al. Proteomics identifies potential immunological drivers of postinfection brain atrophy and cognitive decline. Nature Aging. 2024;4(9):1263‐1278. doi:10.1038/s43587-024-00682-4 39143319 PMC11408246

[76] Hernandez‐Ruiz V , Letenneur L , Fülöp T , et al. Infectious diseases and cognition: do we have to worry? Neurolog Sci. 2022;43(11):6215‐6224. doi:10.1007/s10072-022-06280-9 PMC9305033 35867217

[77] Cinelli M , Quattrociocchi W , Galeazzi A , et al. The COVID‐19 social media infodemic. Sci Rep. 2020;10(1):16598. doi:10.1038/s41598-020-73510-5 33024152 PMC7538912

[78] Gisondi MA , Barber R , Faust JS , et al. A Deadly Infodemic: social Media and the Power of COVID‐19 misinformation. J Med Internet Res. 2022;24(2):e35552. doi:10.2196/35552 35007204 PMC8812140

[79] Crivelli L , Palmer K , Calandri I , et al. Changes in cognitive functioning after COVID‐19: a systematic review and meta‐analysis. Alzheimer's Dement. 2022;18(5):1047‐1066. doi:10.1002/alz.12644 35297561 PMC9073922

[80] Stanek KC , Ones DS . Meta‐analytic relations between personality and cognitive ability. Proc Natl Acad Sci. 2023;120(23):e2212794120. doi:10.1073/pnas.2212794120 37252971 PMC10266031

[81] Bethell J , Andrew MK , Hothi S , Mick P et al. Does social connection mediate the association between neuroticism and cognition? cross‐sectional analysis of the Canadian longitudinal study on aging. Aging Ment Health. 2024;28(3):482‐490. doi:10.1080/13607863.2023.2252369 37667914

[82] Ayalon L , Shiovitz‐Ezra S , Roziner I . A cross‐lagged model of the reciprocal associations of loneliness and memory functioning. Psychol Aging. 2016;31(3):255‐261. doi:10.1037/pag0000075 26974589

[83] Cui M , Liu Y , Yang M , Miura KW , Zhang J , Anme T . Breaking the vicious cycle: reciprocal influences between social isolation and functional disability and the buffering effect of digital inclusion. Arch Gerontol Geriatr. 2025;135:105871. doi:10.1016/j.archger.2025.105871 40294578

[84] McLeod JD . Social stratification and inequality. In: Aneshensel CS , Phelan JC , Bierman A , eds. Handbook of the Sociology of Mental Health. Handbooks of Sociology and Social Research. Springer; 2013:229‐253. doi:10.1007/978-94-007-4276-5

[85] Pearlin LI . The sociological study of stress. J Health Soc Behav. 1989;30(3):241‐256. doi:10.2307/2136956 2674272

[86] Mirowsky J , Ross CE . Social Causes of Psychological Distress. 2nd ed. Aldine De Gruyter; 2003.

[87] Clouston SAP , Link BG . A retrospective on fundamental cause theory: state of the literature and goals for the future. Annu Rev Sociol. 2021;47:1‐26. 10.1146/annurev-soc-090320-094912 PMC8691558 34949900

[88] Latham A , Layton J . Social infrastructure and the public life of cities: studying urban sociality and public spaces. Geography Compass. 2019;13(7):e12444. doi:10.1111/gec3.12444

[89] Mela A , Toldo A . Socio‐Spatial Inequalities in Contemporary Cities. Springer International Publishing; 2019. doi:10.1007/978-3-030-17256-5

[90] Logan JR . Growth, politics, and the stratification of places. Am J Sociol. 1978;84(2):404‐416.

[91] Nutakor JA , Zhou L , Larnyo E , Addai‐Danso S , Tripura D . Socioeconomic status and quality of life: an assessment of the mediating effect of social capital. Healthcare. 2023;11(5):749. doi:10.3390/healthcare11050749 36900754 PMC10001315

[92] Lee S . Education, other socioeconomic indicators, and cognitive function. Am J Epidemiol. 2003;157(8):712‐720. doi:10.1093/aje/kwg042 12697575

[93] Wang X , Bakulski KM , Paulson HL , Albin RL , Park SK . Associations of healthy lifestyle and socioeconomic status with cognitive function in U.S. older adults. Sci Rep. 2023;13(1):7513. doi:10.1038/s41598-023-34648-0 37160962 PMC10170128

[94] Peng S , Perry B . A life course perspective on cognitive aging: the interplay between early and later life stimulating environments. J Health Soc Behav. Published online August 23, 2025. doi:10.1177/00221465251356611 PMC13285902 40847996

[95] Coleman ME , Roessler MEH , Peng S , Roth AR et al. Social enrichment on the job: complex work with people improves episodic memory, promotes brain reserve, and reduces the risk of dementia. Alzheimer's Dement. 2023;19(6):2655‐2665. doi:10.1002/alz.13035 37037592 PMC10272079

[96] Andel R , Veal BM , Howard VJ , MacDonald LA , Judd SE , Crowe M . Retirement and cognitive aging in a racially diverse sample of older Americans. J Am Geriatr Soc. 2023;71(9):2769‐2778. doi:10.1111/jgs.18475 37465869 PMC10526697

[97] Segel‐Karpas D , Ayalon L , Lachman ME . Loneliness and depressive symptoms: the moderating role of the transition into retirement. Aging Ment Health. 2018;22(1):135‐140. doi:10.1080/13607863.2016.1226770 27624519

[98] Donovan NJ , Wu Q , Rentz DM , Sperling RA , Marshall GA , Glymour MM , Glymour MM. loneliness, depression and cognitive function in older U.S. adults. Int J Geriatr Psychiatry. 2017;32(5):564‐573. doi:10.1002/gps.4495 27162047 PMC5102822

[99] Iveniuk J , Piedra LM , Kotwal A , Wilder J , Hawkley L . How race, gender, and cohort shape social isolation and loneliness in Older Americans. J Appl Gerontol. 2026 45(5):951‐962. doi:10.1177/07334648251360449 40717038 PMC12440364

[100] Sheftel MG , Goldman N , Pebley AR , Pratt B , Park SS . Cognitive health disparities by race and ethnicity: the role of occupational complexity and occupational status. Work, Aging Retire. 2024;11(1):64‐78. doi:10.1093/workar/waad023 PMC11634187 39669958

[101] Soh Y , Eng CW , Hodis JD , et al. Racial and ethnic differences in occupational complexity and late‐life cognition. Alzheimer's Dement. 2022;18(suppl 11):Se063760. doi:10.1002/alz.063760

[102] Miyawaki CE . Association of social isolation and health across different racial and ethnic groups of older Americans. Ageing Soc. 2015;35(10):2201‐2228. doi:10.1017/S0144686X14000890 26494934 PMC4610249

[103] Umberson D , Lin Z , Cha H . Gender and social isolation across the life course. J Health Soc Behav. 2022;63(3):319‐335. doi:10.1177/00221465221109634 35856404 PMC10409601

[104] Evans IEM , Martyr A , Collins R , Brayne C , Clare L . Social isolation and cognitive function in later life: a systematic review and meta‐analysis. J Alzheimers. 2019;70(s1):S119‐S144. doi:10.3233/JAD-180501 PMC6700717 30372678

[105] Lin Z , Joyner K , Manning WD . Sexual orientation and social isolation from early adulthood to early midlife. J Health Soc Behav. Published online June 16, 2025. doi:10.1177/00221465251340020 PMC13219788 40518989

[106] Vasen E , Poteat T , Maman S . A review of psychosocial interventions to improve parental support of transgender youth. Transgender Health. Published online February 3, 2025. doi:10.1089/trgh.2024.0139

[107] Tran NM , McKay T , Gonzales G , Dusetzina SB , Fry C . Aging in isolation: sexual orientation differences in navigating cognitive decline. SSM—Population Health. 2024;27:101699. doi:10.1016/j.ssmph.2024.101699 39139827 PMC11320599

[108] Small ML , Adler L . The role of space in the formation of social ties. Annu Rev Sociol. 2019;45(1):111‐132. doi:10.1146/annurev-soc-073018-022707

[109] Latham A , Layton J . Social infrastructure: why it matters and how urban geographers might study it. Urban Geography. 2022;43(5):659‐668. doi:10.1080/02723638.2021.2003609

[110] Klinenberg E . Palaces for the People: How Social Infrastructure can Help Fight Inequality, Polarization, and the Decline of Civic Life. Crown Publishers; 2018.

[111] Layton J , Latham A . Social infrastructure and public life – notes on Finsbury Park, London. Urban Geography. 2022;43(5):755‐776. doi:10.1080/02723638.2021.1934631

[112] Clarke PJ , Ailshire JA , House JS , Morenoff JD , King K , Melendez R , Langa KM Cognitive function in the community setting: the neighbourhood as a source of ‘cognitive reserve’? J Epidemiol Community Health. 2012;66(8):730‐736. doi:10.1136/jech.2010.128116 21515547 PMC3387518

[113] Finlay J , Esposito M , Langa KM , Judd S , Clarke P . Cognability: an ecological theory of neighborhoods and cognitive aging. Soc Sci Med. 2022;309:115220. doi:10.1016/j.socscimed.2022.115220 35926362 PMC9661364

[114] Finlay J , Westrick AC , Guzman V , Meltzer G . Neighborhood built environments and health in later life: a literature review. J Aging Health. 2025;37(1‐2):3‐17. doi:10.1177/08982643231217776 37994863 PMC11111591

[115] Walkability YuCYN , Third place engagement, and their impact on physical activity and social capital for older adults living alone and with others. J Aging Health. 2026;38(3‐4):192‐199. doi:10.1177/08982643251323301 39992053

[116] CDC Data Shows Over 70 Million U.S. Adults Reported Having a Disability . Centers for Disease Control and Prevention; 2024. https://www.cdc.gov/media/releases/2024/s0716‐Adult‐disability.html

[117] Carpiano RM . Toward a neighborhood resource‐based theory of social capital for health: can Bourdieu and sociology help? Soc Sci Med. 2006;62(1):165‐175. doi:10.1016/j.socscimed.2005.05.020 15992978

[118] Chetty R , Friedman J , Hendren N , Jones M , Porter S . The Opportunity Atlas: Mapping the Childhood Roots of Social Mobility. National Bureau of Economic Research; 2018. Working paper 25147. doi:10.3386/w25147

[119] Ziersch AM , Baum FE , MacDougall C , Putland C . Neighbourhood life and social capital: the implications for health. Soc Sci Med. 2005;60(1):71‐86. doi:10.1016/j.socscimed.2004.04.027 15482868

[120] Ross CE , Jang SJ . Neighborhood disorder, fear, and mistrust: the buffering role of social ties with neighbors. Am J Community Psychol. 2000;28(4):401‐420. doi:10.1023/A:1005137713332 10965384

[121] Cullen FT . Challenging Criminological Theory: The Legacy of Ruth Rosner Kornhauser. Cullen FT , Wilcox P , Sampson RJ , Dooley BD , eds. 1st ed. Routledge; 2017. doi:10.4324/9781315081601

[122] Bloom ND . The Great American Transit Disaster: A Century of Austerity, Auto‐Centric Planning, and White Flight. University Of Chicago Press; 2023.

[123] Phillips NE , Levy BL , Sampson RJ , Small ML , Wang RQ . The social integration of American cities: network measures of connectedness based on everyday mobility across neighborhoods. Sociol Methods Res. 2021;50(3):1110‐1149. doi:10.1177/0049124119852386

[124] Roberto E . The spatial proximity and connectivity method for measuring and analyzing residential segregation. Sociol Methodol. 2018;48(1):182‐224. doi:10.1177/0081175018796871

[125] Andrews R , Casey M , Hardy BL , Logan TD . Location matters: historical racial segregation and intergenerational mobility. Economics Letters. 2017;158:67‐72. doi:10.1016/j.econlet.2017.06.018

[126] Glenn ND . Distinguishing age, period, and cohort effects. In: Mortimer JT , Shanahan MJ , eds. Handbook of the Life Course. Kluwer‐Plenum; 2004:465‐476.

[127] Ryder NB . The cohort as a concept in the study of social change. Am Sociol Rev. 1965;30(6):843‐861. 5846306

[128] Yang YC . Aging, cohorts, and methods. In: Binstock RH , George LK , eds. Handbook of Aging and the Social Sciences. 7th ed. Handbooks of aging. Elsevier/Academic Press; 2011:17‐30.

[129] Umberson D , Donnelly R . Social isolation: an unequally distributed health hazard. Annu Rev Sociol. 2023;49(1):379‐399. doi:10.1146/annurev-soc-031021-012001 38106980 PMC10722883

[130] Cornwell B . Age trends in daily social contact patterns. Res Aging. 2011;33(5):598‐631. doi:10.1177/0164027511409442

[131] Peng S , Roth AR , Perry BL . Social connections over the life course: appreciating distinct dimensions of social connections. Res Aging. 2025;48(5‐6):271‐285. doi:10.1177/01640275251377777 40911374 PMC12462710

[132] Cornwell B , Laumann EO , Schumm LP . The social connectedness of older adults: a national profile. Am Sociol Rev. 2008;73(2):185‐203. doi:10.1177/000312240807300201 19018292 PMC2583428

[133] Zeng Y , Chen YC , Lum TYS . Longitudinal impacts of grandparent caregiving on cognitive, mental, and physical health in China. Aging Ment Health. 2021;25(11):2053‐2060. doi:10.1080/13607863.2020.1856779 33291945

[134] Bordone V , Arpino B . Do grandchildren influence how old you feel? J Aging Health. 2016;28(6):1055‐1072. doi:10.1177/0898264315618920 26656157

[135] Kim HJ , Kang H , Johnson‐Motoyama M . The psychological well‐being of grandparents who provide supplementary grandchild care: a systematic review. J Fam Stud. 2017;23(1):118‐141. doi:10.1080/13229400.2016.1194306

[136] Silverstein M , Zuo D . Grandparents caring for grandchildren in rural China: consequences for emotional and cognitive health in later life. Aging Ment Health. 2021;25(11):2042‐2052. doi:10.1080/13607863.2020.1852175 33251822

[137] Hwang TJ , Rabheru K , Peisah C , Reichman W , Ikeda M . Loneliness and social isolation during the COVID‐19 pandemic. Int Psychogeriatr. 2020;32(10):1217‐1220. doi:10.1017/S1041610220000988 32450943 PMC7306546

[138] Liu H , Umberson DJ . The times they are a changin’: marital status and health differentials from 1972 to 2003. J Health Soc Behav. 2008;49(3):239‐253. doi:10.1177/002214650804900301 18771061 PMC3150568

[139] Putnam R . Bowling Alone: The Collapse and Revival of American Community. Simon and Schuster; 2000.

[140] Liu J . An epidemic of social isolation? age and cohort trends of social connectedness among older adults, 2004‐2018. Soc Sci Res. 2025;131:103212. doi:10.1016/j.ssresearch.2025.103212

[141] Ang S . Life course social connectedness: age‐cohort trends in social participation. Adv in Life Course Res. 2019;39:13‐22. doi:10.1016/j.alcr.2019.02.002

[142] Roth AR , Peng S , Perry BL . Personal network bridging potential across geographic context. J Gerontol Ser B. 2022;77(3):626‐635. doi:10.1093/geronb/gbab103 PMC8893139 34097016

[143] Burt CJ , Kondo MC , Hohl BC , et al. Community greening, fear of crime, and mental health outcomes. Am J Community Psychol. 2022;69(1‐2):46‐58. doi:10.1002/ajcp.12544 34333789 PMC8803989

[144] Chayko M . Techno‐social life: the internet, digital technology, and social connectedness. Sociol Compass. 2014;8(7):976‐991. doi:10.1111/soc4.12190

[145] Chayko M . Superconnected: The Internet, Digital Media, and Techno‐Social Life. 3rd ed. SAGE Publications, Inc.; 2021.

[146] Shanmugasundaram M , Tamilarasu A . The impact of digital technology, social media, and artificial intelligence on cognitive functions: a review. Frontiers in Cognition. 2023;2:1203077. doi:10.3389/fcogn.2023.1203077

[147] Pentina I , Xie T , Hancock T , Bailey A . Consumer‐machine relationships in the age of artificial intelligence: systematic literature review and research directions. Psychol & Marketing. 2023;40(8):1593‐1614. doi:10.1002/mar.21853

[148] Chetty R , Jackson MO , Kuchler T , et al. Social capital I: measurement and associations with economic mobility. Nature. 2022;608(7921):108‐121. doi:10.1038/s41586-022-04996-4 35915342 PMC9352590

[149] Ang S , Chen TY . Going online to stay connected: online social participation buffers the relationship between pain and depression. J Gerontol Ser B. 2019;74(6):1020‐1031. doi:10.1093/geronb/gby109 30260444

[150] Yu X , Ang S , Zhang Y . Exploring rural‐urban differences in the association between internet use and cognitive functioning among older adults in China. J Gerontol B Psychol Sci Soc Sci. 2024;79(4):gbad195. doi:10.1093/geronb/gbad195 38147307

[151] Benge JF , Scullin MK . A meta‐analysis of technology use and cognitive aging. Nat Hum Behav. 2025;9(7):1405‐1419. doi:10.1038/s41562-025-02159-9 40229575 PMC12333551

[152] Capraro V , Globig LK , Rausch Z , et al. A consensus statement on potential negative impacts of smartphone and social media use on adolescent mental health. Seton Hall Law School Legal Studies Research. SSRN. Forthcoming 2025. doi:10.2139/ssrn.5256747

[153] Rodriguez M , Schertz KE , Kross E . How people think about being alone shapes their experience of loneliness. Nat Commun. 2025;16(1):1594. doi:10.1038/s41467-025-56764-3 39939585 PMC11821820

[154] Whitfield T , Barnhofer T , Acabchuk R , et al. The effect of mindfulness‐based programs on cognitive function in adults: a systematic review and meta‐analysis. Neuropsychol Rev. 2022;32(3):677‐702. doi:10.1007/s11065-021-09519-y 34350544 PMC9381612

[155] Finlay J , Franke T , McKay H , Sims‐Gould J . Therapeutic landscapes and wellbeing in later life: impacts of blue and green spaces for older adults. Health Place. 2015;34:97‐106. doi:10.1016/j.healthplace.2015.05.001 25982704

[156] Fernández A , Lu Y , Chopik WJ , Harari GM , Rhee L , Bayer JB . The right fit: when socially anxious individuals gain the most from social interactions. Soc Psychol Personal Sci. Published online February 9, 2026. doi:10.1177/19485506261416010

[157] Smith‐Greenaway E , Verdery AM , Carr D . The new sociology of bereavement. Annu Rev Sociol. 2025;51:357‐375. 40880607 10.1146/annurev-soc-090324-035534 PMC12383204

[158] Békés V , Roberts K , Németh D . Competitive neurocognitive processes following bereavement. Brain Res Bull. 2023;199:110663. doi:10.1016/j.brainresbull.2023.110663 37172799

[159] Pérez HCS , Ikram MA , Direk N , Tiemeier H . Prolonged grief and cognitive decline: a prospective population‐based study in middle‐aged and older persons. Am J Geriatr Psychiatry. 2018;26(4):451‐460. doi:10.1016/j.jagp.2017.12.003 29329723

[160] Johnson LA , Melendez C , Burton R , Sheikh N , Clarkson G . An examination of cognitive function abilities in bereaved adults. Am Jl Hosp Palliat Care. 2024;41(3):324‐328. doi:10.1177/10499091231204868 37787947

[161] Tolkamp M , Pollmann‐Schult M . Widowhood and loneliness: do close relations with adult children alleviate loneliness among widowed parents? Aging Ment Health. 2025;29(12):2229‐2239. doi:10.1080/13607863.2025.2512214 40658960

[162] Verdery AM , Smith‐Greenaway E , Margolis R , Daw J . Tracking the reach of COVID‐19 kin loss with a bereavement multiplier applied to the United States. Proc Natl Acad Sci. 2020;117(30):17695‐17701. doi:10.1073/pnas.2007476117 32651279 PMC7395491

[163] Garcia MA , Needham BL , Goosby BJ , Hummer RA , Liu H , Umberson D . Death of a parent, racial inequities, and cardiovascular disease risk in early to mid‐adulthood. J Health Soc Behav. 2025;66(2):165‐181. doi:10.1177/00221465241273870 39367799 PMC11971391

[164] Umberson D , Olson JS , Crosnoe R , Liu H , Pudrovska T , Donnelly R . Death of family members as an overlooked source of racial disadvantage in the United States. Proc Natl Acad Sci USA. 2017;114(5):915‐920. doi:10.1073/pnas.1605599114 28115712 PMC5293066

[165] Doryab A , Villalba DK , Chikersal P , et al. Identifying behavioral phenotypes of loneliness and social isolation with passive sensing: statistical analysis, data mining and machine learning of smartphone and Fitbit data. JMIR mHealth and uHealth. 2019;7(7):e13209. doi:10.2196/13209 31342903 PMC6685126

[166] Qirtas MM , Pesch D , Zafeiridi E , White EB . Privacy preserving loneliness detection: a federated learning approach: Proceedings of the 2022 IEEE International Conference on Digital Health (ICDH), 157‐162. IEEE; 2022:doi:10.1109/ICDH55609.2022.00032

[167] Prabhu D , Kholghi M , Sandhu M , et al. Sensor‐based assessment of social isolation and loneliness in older adults: a survey. Sensors. 2022;22(24):9944. doi:10.3390/s22249944 36560312 PMC9781772

[168] Krause N , Chatters LM , Meltzer T , Morgan DL . Negative interaction in the church: insights from focus groups with older adults. Rev Relig Res. 2000;41(4):510. doi:10.2307/3512318

[169] Krause N , Chatters LM , Meltzer T , Morgan DL . Using focus groups to explore the nature of prayer in late life. J Aging Stud. 2000;14(2):191‐212. doi:10.1016/S0890-4065(00)80011-0

[170] Kyne D , Aldrich DP . Capturing bonding, bridging, and linking social capital through publicly available data. RHCPP. 2020;11(1):61‐86. doi:10.1002/rhc3.12183

[171] Aruqaj B . An integrated approach to the conceptualisation and measurement of social cohesion. Soc Indic Res. 2023;168(1‐3):227‐263. doi:10.1007/s11205-023-03110-z PMC10212225 37362178

[172] Martin CL , Ward‐Caviness CK , Dhingra R , Zikry TM et al. Neighborhood environment, social cohesion, and epigenetic aging. Aging. 2021;13(6):7883‐7899. doi:10.18632/aging.202814 33714950 PMC8034890

[173] Choi J , Han SH , Ng YT , Muñoz E . Neighborhood cohesion across the life course and effects on cognitive aging. J Gerontol Ser B. 2023;78(10):1765‐1774. doi:10.1093/geronb/gbad095 PMC10561885 37350749

[174] Kang W . Psychological distress mediates the associations between neighborhood social cohesion (NSC) and cognitive performance in older adults. Curr Psychol 2024;43(8):7144‐7152. doi:10.1007/s12144-023-04887-5

